# Antimicrobial stewardship in neonatal intensive care units in resource-limited regions of China: a protocol for a multicenter, before-and-after, interrupted time series quality improvement study

**DOI:** 10.3389/fpubh.2026.1837412

**Published:** 2026-06-02

**Authors:** Bo Wang, Weibing Qiu, Min He, Yang Wang, Junxue Zhu, Yan Hu, Jia Zhang

**Affiliations:** 1 Department of Pediatrics, Suqian First Hospital, Suqian, China; 2 Department of Neonatology, Siyang Hospital, Suqian, China; 3 Department of Neonatology, Sihong First Hospital, Suqian, China; 4 Department of Pediatrics, The Affiliated Huaihai Hospital of Xuzhou Medical University, Xuzhou, China; 5 Department of Neonatology, Sihong Hospital, Suqian, China; 6 Department of Neonatology, Shuyang Hospital of Traditional Chinese Medicine (Clinical College of Yangzhou University Medical College), Suqian, Jiangsu, China

**Keywords:** antimicrobial stewardship, interrupted time series analysis, neonatal intensive care unit, quality improvement, resource-limited settings

## Abstract

**Introduction:**

Antibiotic overuse is prevalent in neonatal intensive care units (NICUs) and is associated with adverse neonatal outcomes and antimicrobial resistance. While antimicrobial stewardship programs (ASPs) have been implemented globally, antibiotic utilization rates remain high in China, particularly in resource-limited regional settings. Evidence indicates that guideline dissemination alone is insufficient to alter established prescribing behaviors, underscoring the need for pragmatic, system-level quality improvement (QI) strategies. This study outlines a multicenter QI initiative designed to optimize antibiotic use in NICUs within a resource-limited region of China.

**Methods:**

This ongoing study uses a multicenter, before-and-after interrupted time series (ITS) design across five NICUs in the Suqian region. A bundled ASP intervention is being implemented, comprising standardized early-onset sepsis risk stratification, mandatory 48–72-h antibiotic reassessment, protocolized empirical antibiotic regimens, and a two-tier governance structure supported by monthly Plan–Do–Study–Act cycles. Monthly aggregated data are collected during a 12-month baseline period (2025) and a 12-month intervention period (2026–2027). Primary outcomes are the antibiotic use rate and the antibiotic utilization rate (days of therapy per 1,000 patient-days). Balancing measures include the incidence of late-onset sepsis, in-hospital mortality, and antibiotic re-initiation rate. Process adherence is monitored as a key implementation indicator. Segmented regression ITS analysis and statistical process control charts are used to evaluate the intervention effect.

**Discussion:**

This study evaluates a pragmatic, workflow-integrated ASP QI model for regional NICUs with limited resources. By integrating standardized clinical pathways, structured reassessment, and continuous data-driven feedback within a multicenter collaborative framework, the intervention aims to safely reduce unnecessary antibiotic exposure. The findings will provide evidence to inform scalable antimicrobial stewardship strategies in similar resource-constrained neonatal care settings.

**Clinical trial registration:**

https://www.chictr.org.cn/, dentifier: ChiCTR2600120301.

## 1 Introduction

Antibiotics are among the most frequently prescribed medications in neonatal intensive care units (NICUs) (1). However, substantial evidence consistently indicates widespread overuse of antibiotics in NICU clinical practice (2, 3). Unnecessary antibiotic exposure is increasingly linked to adverse neonatal outcomes, including necrotizing enterocolitis (NEC), late-onset sepsis (LOS), and multidrug-resistant organisms (4–8). Consequently, optimizing antibiotic use in NICUs has become an important global public health priority to improve neonatal outcomes (9).

Despite the gradual implementation of antimicrobial stewardship programs (ASPs) worldwide, marked variations in antibiotic use persist across countries and regions, with particularly high levels reported in China (10). In some high-income countries, antibiotic utilization rates (AURs) in NICUs have declined to approximately 250 days of therapy (DOTs) per 1,000 patient-days (11). In contrast, recent multicenter surveys in China have reported median NICU AURs of 500–530 DOTs per 1,000 patient-days in regions such as Hunan and Jiangsu provinces, substantially exceeding levels reported in many high-income settings (12–14). The Suqian region, located in northern Jiangsu Province, has relatively limited healthcare resources and subspecialty development compared with more developed urban centers within the province. Our baseline assessment demonstrated an AUR of 636 DOTs per 1,000 patient-days in NICUs in the Suqian region in 2024, indicating a particularly high burden of antibiotic use. Therefore, conducting an antimicrobial stewardship intervention in this region holds significant implications for exploring rational antibiotic use pathways in NICUs within resource-limited settings.

Previous studies have shown that the dissemination of clinical guidelines alone or passive educational interventions is often insufficient to change long-established prescribing behaviors among physicians (15). Increasing evidence suggests that systematic quality improvement (QI) strategies are required to achieve sustained practice change (16). Systematic reviews and meta-analyses have demonstrated that QI-based interventions can effectively reduce antibiotic use in NICUs without increasing neonatal mortality or other serious adverse outcomes, supporting the safety and potential benefits of such approaches (17). Nevertheless, directly transferring well-established QI models from other institutions to regional or resource-limited hospital systems remains challenging. Barriers include constrained staffing resources, differences in safety culture (such as defensive medical practices), and locally specific antimicrobial resistance epidemiology (18, 19). Accordingly, there is a clear need to develop QI strategies that are pragmatic, sustainable, and readily integrated into routine clinical workflows within local practice contexts, thereby narrowing the gap between guideline recommendations and real-world clinical practice.

Against this background, the present study aims to evaluate the effectiveness of a regional, multicenter QI program in reducing antibiotic use in NICUs. Using a before-and-after interrupted time series (ITS) design, this study will implement a bundled intervention across five NICUs in the Suqian region, incorporating infection risk stratification, mandatory 48-h antibiotic reassessment, and a two-tier governance structure. The impact of this intervention on antibiotic utilization and associated clinical outcomes will be systematically assessed.

## 2 Methods

### 2.1 Study design

This multicenter QI initiative utilizes a before-and-after ITS design to evaluate changes in antibiotic use and clinical outcomes in Suqian NICUs. Positioned as a practice-oriented initiative, it focuses on assessing the clinical effectiveness of integrating interventions into routine workflows rather than implementation mechanisms.

The design, implementation, and reporting of this study follow the Standards for Quality Improvement Reporting Excellence (SQUIRE) 2.0 guidelines (20). The study protocol was approved by the Ethics Committee of the Suqian Municipal Science and Technology Bureau (approval number: 2025-SR-0023). As this project constitutes a system-level QI initiative aimed at process optimization, a waiver of informed consent was granted by the ethics committee. All patient data are de-identified to ensure confidentiality. The study has been registered with the Chinese Clinical Trial Registry (registration number: ChiCTR2600120301).

### 2.2 Study Period

The study comprises three consecutive phases:

**1) Baseline period (January 2025 to December 2025):**

During the baseline period, all participating centers followed their usual clinical practices for the diagnosis and management of neonatal infections. Data from this period were used to assess baseline antibiotic use.

**2) Preparation period (January 2026 to March 2026):**

This phase encompasses baseline data analysis, investigation of drivers of antibiotic use, development of the intervention package, staff training, and establishment of a multicenter data coordination mechanism.

**3) Intervention period (April 2026 to March 2027):**

The antimicrobial stewardship QI intervention is scheduled to be implemented simultaneously across all participating centers, with ongoing monitoring of relevant outcome and process indicators.

### 2.3 Study setting and participants

This study is conducted concurrently across five NICUs in the Suqian region: Suqian First Hospital, Sihong Hospital, Sihong First Hospital, Shuyang Hospital of Traditional Chinese Medicine, and Siyang Hospital. These institutions serve as the primary referral centers for neonatal care within the region. The annual number of NICU discharges for each participating center in 2024 is presented in Table 1.

**Table 1:** Characteristics of participating neonatal intensive care units in 2024.

Institution	Hospital level	Annual NICU admissions
Suqian First Hospital	Tertiary	862
Sihong Hospital	Tertiary	524
Sihong First Hospital	Secondary	395
Shuyang Hospital of Traditional Chinese Medicine	Tertiary	872
Siyang Hospital	Tertiary	484

#### Inclusion criteria

1. All neonates admitted to the NICUs of participating centers during the study period;
2. Admission occurring within either the baseline or intervention period.

#### Exclusion criteria

1. Neonates with a confirmed diagnosis of severe infection prior to admission who had already received systemic antibiotic therapy;
2. Neonates with missing key clinical data that precluded evaluation of the primary outcome measures.

### 2.4 Sample size considerations

As this study is a QI initiative with monthly aggregated data as the primary unit of analysis, no formal sample size or power calculation based on individual-level hypotheses was performed. The study design includes 12 consecutive monthly data points before and after the intervention. Methodological literature indicates that ITS analyses typically require a minimum of 8–10 data points in both the pre- and post-intervention phases to establish stable baseline trends and detect meaningful changes over time (21, 22). Accordingly, the planned study design is considered sufficient to support statistical process control analyses and ITS regression modeling.

### 2.5 Baseline assessment and root cause analysis

Before developing the intervention, we conducted a structured baseline assessment using 2025 data, informed by multidisciplinary discussions with NICU staff to identify drivers of high antibiotic use.

Through process mapping and multidisciplinary review, several key factors contributing to antibiotic overuse were identified, including:

1. Lack of standardized criteria for initiating antibiotic therapy;
2. Risk-averse approach to early-onset sepsis (EOS) risk assessment;
3. Absence of a systematic 48–72-h antibiotic reassessment process;
4. Insufficient monitoring and feedback mechanisms related to antibiotic use.

**Figure 1:**
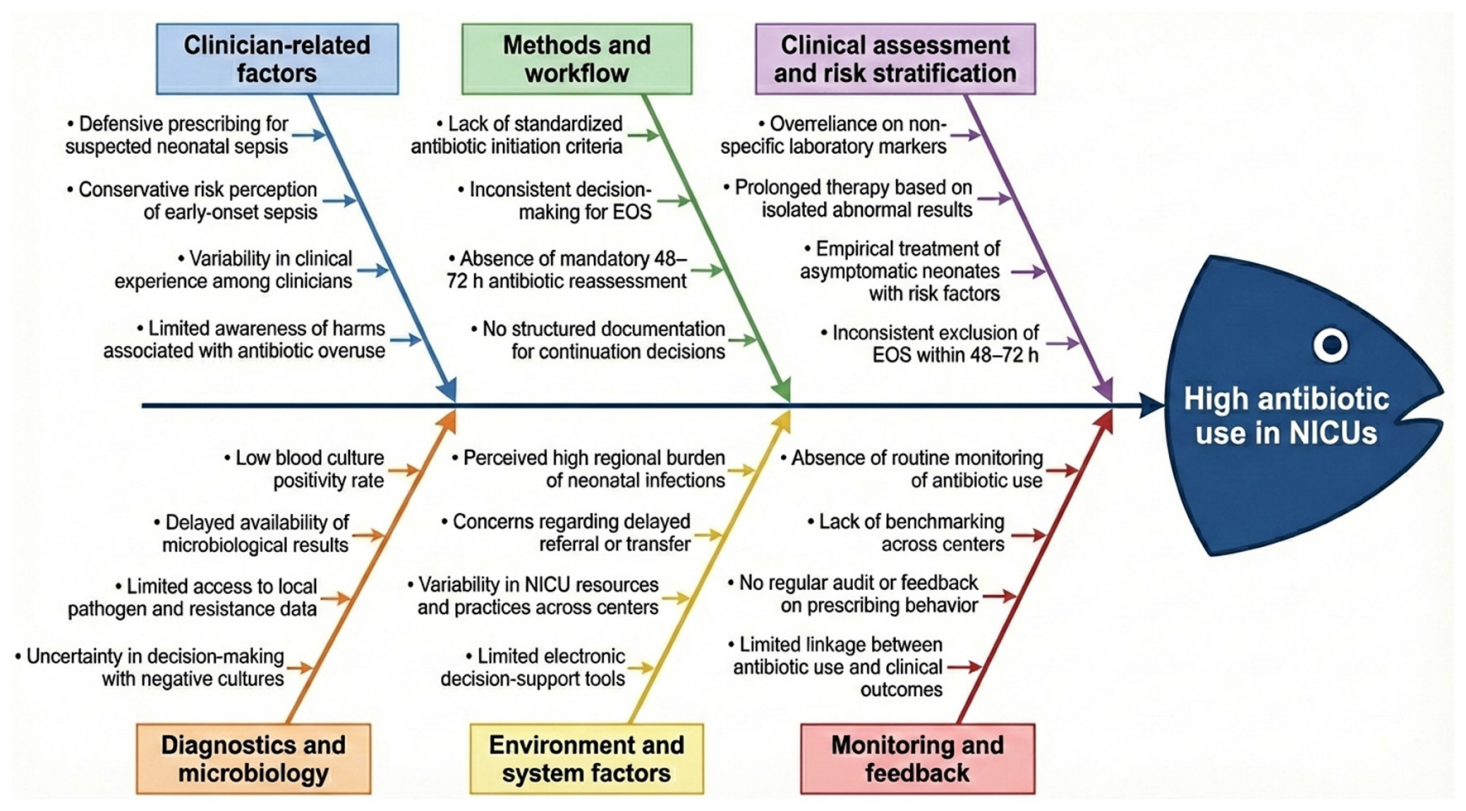
Fishbone diagram illustrating key contributors to high antibiotic use in NICUs in the Suqian region. NICU, Neonatal Intensive Care Unit; EOS, early-onset sepsis; AUR, antibiotic utilization rate.

These contributing factors were synthesized into a fishbone diagram (Figure 1) and served as the conceptual framework for the design of the antimicrobial stewardship QI intervention bundle.

### 2.6 Intervention

The antimicrobial stewardship intervention implemented in this study consists of a multicomponent clinical QI bundle, with the core objective of optimizing decision-making processes related to antibiotic initiation, reassessment, and discontinuation.

#### 2.6.1 Module 1. establishment of a two-tier QI team structure

1) Regional steering committee

A regional steering committee was established and led by Suqian First Hospital. The committee comprises department directors from each participating center, directors of pharmacy services, infection control leads, and the principal investigators. The committee is responsible for developing unified standards, organizing training activities, coordinating data collection, and convening monthly review meetings.

2) Hospital-level core implementation teams

Each participating hospital established a core implementation team consisting of the NICU director (team lead), one to two senior attending neonatologists (designated liaisons), a clinical pharmacist, an infection control nurse, and a microbiology laboratory representative. These teams are responsible for local implementation of the intervention, data collection, routine audit, and feedback within their respective NICUs.

3) Multicenter coordination mechanism

All centers submit monthly data on process adherence to the regional steering committee and participate in cross-center discussions and feedback during monthly collaborative meetings. Each center designates fixed personnel responsible for overseeing antimicrobial stewardship processes. Cases deviating from the intervention protocol are explicitly flagged in monthly data reports and reviewed during collaborative meetings. Continuous monitoring of key process indicators promotes consistent implementation across centers.

The two-tier implementation structure is designed to minimize additional workload. Hospital-level core teams are composed of a small number of key personnel whose existing responsibilities already include clinical leadership, pharmacy, or infection control. Frontline clinicians are primarily engaged through the standardized clinical pathway at the point of care rather than through frequent additional meetings. Coordination, data aggregation, and feedback responsibilities are centralized within the hospital-level core team and the regional steering committee, whose monthly meetings are intended to consolidate previously unstructured, *ad hoc* communications into a more efficient feedback mechanism.

#### 2.6.2 Module 2. standardization of antibiotic initiation criteria for EOS

**Figure 2:**
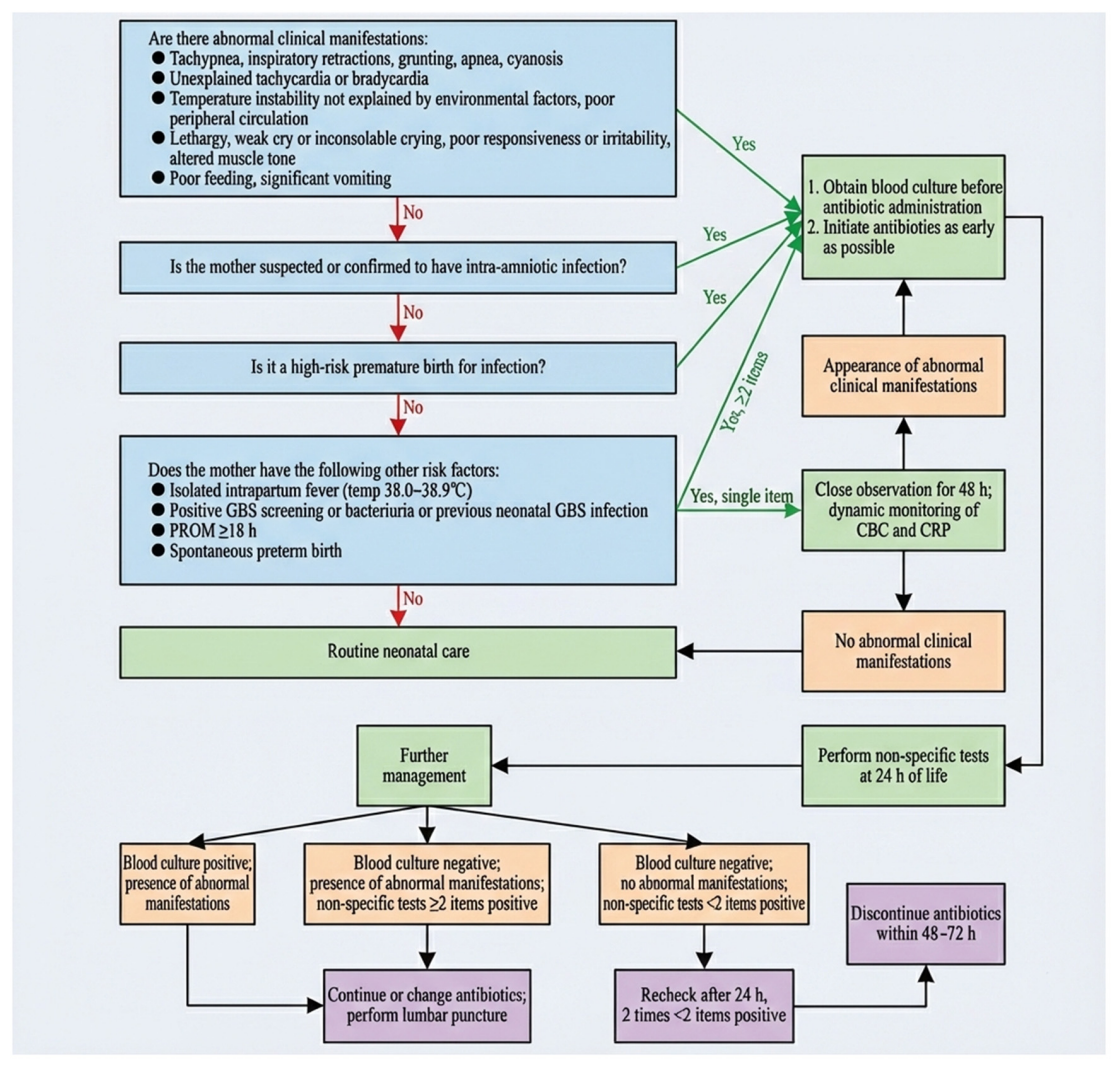
Standardized clinical pathway for risk assessment and antibiotic management of suspected early-onset sepsis. 1. Suspected intra-amniotic infection: Defined as maternal intrapartum fever (temperature ≥39.0 °C or 38.0 °C−38.9 °C persisting for >30 min) accompanied by one or more of the following clinical signs: (1) maternal leukocytosis (>15 × 10^9^/L); (2) purulent cervical discharge; (3) fetal tachycardia (>160 beats/min) persisting for 10 min or longer; (4) cloudy or foul-smelling amniotic fluid. 2. Confirmed intra-amniotic infection: defined as maternal intrapartum fever (temperature ≥39.0 °C or 38.0 °C−38.9 °C persisting for >30 min) accompanied by one or more of the following positive laboratory findings: (1) positive Gram stain of amniotic fluid; (2) positive amniotic fluid culture; (3) histopathological evidence of inflammation or infection in the umbilical cord, placenta, or fetal membranes. 3. Preterm infants at high risk for infection: defined as: (1) Infants with gestational age ≥35 weeks and ≥1 perinatal risk factor(s): ① intra-amniotic infection (confirmed or suspected); ② PROM ≥18 h. (2) Infants with gestational age < 35 weeks and ≥1 perinatal risk factor(s): ① intra-amniotic infection (confirmed or suspected); ② preterm birth caused by PROM. GBS, group B streptococcus; PROM, premature rupture of membranes; CBC, complete blood count; CRP, C-reactive protein.

To minimize inter-center and inter-physician variability in EOS management, a unified *EOS Risk Assessment and Antibiotic Management Clinical Pathway* (Figure 2) was developed based on nationally published evidence-based recommendations in China (23) and will be mandatorily implemented across all participating NICUs. This pathway translates complex clinical decision-making into a stepwise, standardized workflow. Core assessment domains include the following:

**1) Maternal perinatal infection risk factors**

Including, but not limited to:

- Suspected or confirmed intra-amniotic infection (definitions provided in the Figure 2 legend) (24);
- Prolonged rupture of membranes ≥18 h;
- Spontaneous preterm birth or preterm birth of unclear etiology;
- Maternal Group B Streptococcus colonization or bacteriuria;
- Isolated intrapartum maternal fever.

These factors are considered to increase EOS risk but do not independently constitute absolute indications for empirical antibiotic therapy.

**2) High-risk preterm infants and neonatal clinical presentation**

A distinction is made between high-risk preterm infants and non-specific clinical signs:

- High-risk preterm infants constitute an indication for initiating antibiotic therapy (definitions provided in the Figure 2 legend) (23);
- Severe abnormal clinical manifestations (e.g., septic shock, requirement for cardiopulmonary resuscitation, need for invasive mechanical ventilation within the first 3 days of life or following clinical deterioration, severe apnea) are considered absolute indications for antibiotic initiation;
- Other nonspecific clinical abnormalities require integrated assessment incorporating perinatal risk factors and laboratory findings.

**3) Laboratory findings**

Non-specific laboratory markers (WBC count, I/T ratio, CRP, PCT, PLT) are used as adjunctive tools. Abnormality in a single parameter alone does not constitute sufficient justification for initiating or prolonging antibiotic therapy. Definitions of laboratory positivity are provided in Supplementary Table S1 (Supplemental material 1).

The pathway will be embedded into routine workflows as a standardized form. Documentation will be mandatory for all empirical antibiotic initiations. Deviations from pathway recommendations require explicit documentation of clinical justification in the medical record. Pathway adherence is monitored as a core process indicator and incorporated into monthly quality feedback and Plan-Do-Study-Act (PDSA) cycles.

#### 2.6.3 Module 3. Mandatory 48–72-h antibiotic reassessment

All neonates receiving antibiotics undergo mandatory reassessment at 48–72 h, covering:

1. Changes in clinical status;
2. Microbiological test results;
3. Dynamic trends in nonspecific laboratory markers.

Based on reassessment findings, clinicians must select one of the following actions:

1. Discontinue antibiotics;
2. Modify or de-escalate the antibiotic regimen;
3. Continue therapy with explicit documentation of clinical justification.

For neonates with suspected EOS in whom the diagnosis is excluded within 48–72 h, discontinuation of antibiotics is mandatory (see Figure 2). All antibiotic-treated neonates must complete reassessment and a documented treatment decision within the 48–72-h window. Failure to complete reassessment is classified as a protocol deviation and is identified during monthly audits and targeted for subsequent improvement efforts. Diagnostic criteria for EOS and LOS are provided in Supplementary Table S2 (Supplementary material 1).

#### 2.6.4 Module 4. Standardization of empirical antibiotic regimens

During the intervention period, empirical antibiotic prescribing follows unified principles across centers:

1. EOS: ampicillin (or penicillin) in combination with a third-generation cephalosporin;
2. Community-acquired LOS: preferential monotherapy with a third-generation cephalosporin;
3. Hospital-acquired LOS: selection guided by local microbiological profiles and resistance patterns, with preference for narrow-spectrum combination regimens.

Once pathogen identification and susceptibility results become available, antibiotic therapy is promptly narrowed to targeted, susceptible agents, with monotherapy preferred whenever feasible.

#### 2.6.5 Module 5. Staff education and decision support

During both the preparation and intervention phases, targeted educational sessions, visual workflow displays, and concise decision-support materials are provided to enhance NICU healthcare workers' awareness of appropriate antibiotic use and the goals of the QI initiative.

#### 2.6.6 Module 6. Continuous monitoring and adaptive optimization using PDSA cycles

To ensure consistent implementation across multiple centers and enable dynamic optimization based on real-time feedback, a structured “monitor–feedback–improve” loop is established. Rapid PDSA cycles are embedded within the two-tier governance structure to drive continuous QI.

PDSA cycles are integrated into the monthly multicenter collaborative meetings as follows:

1. Plan: During each monthly meeting convened by the regional steering committee, 1–2 high-priority, actionable problems (e.g., delayed antibiotic discontinuation) are identified based on data review, and targeted improvement plans are formulated;
2. Do: Hospital-level implementation teams execute the improvement plan within their NICUs over the subsequent monthly cycle;
3. Study: Relevant process data (e.g., audit records for antibiotic courses exceeding 72 h) are collected and reported at the next collaborative meeting, with cross-center comparison;
4. Act: The steering committee and participating centers jointly evaluate effectiveness. Successful practices are incorporated into standardized operating procedures and disseminated across all centers; ineffective strategies are revised, and a subsequent PDSA cycle is initiated.

### 2.7 Outcomes

#### 2.7.1 Primary outcomes

1. Antibiotic use rate: the proportion of neonates who received at least one course of systemic antibiotic therapy during the study period;
2. AUR: defined as the number of antibiotic days per 1,000 patient-days.

#### 2.7.2 Balancing measures

1. Incidence of LOS: the number of sepsis cases diagnosed ≥72 h after birth based on positive blood cultures and/or compatible clinical syndrome, divided by the total number of NICU admissions during the same period and expressed as a percentage;
2. All-cause in-hospital NICU mortality;
3. Antibiotic re-initiation rate: re-initiation of systemic antibiotics within 3 days after antibiotic discontinuation.

#### 2.7.3 Process measures

1. Intervention adherence rate: the proportion of eligible cases in which both antibiotic initiation assessment and the mandatory 48–72-h reassessment were completed as specified by the intervention protocol.

### 2.8 Data collection and management

To ensure data quality and comparability, standardized collection and reporting procedures will be coordinated by the regional steering committee.

#### 2.8.1 Data elements and sources

All participating centers collect data across three domains:

1. Baseline data: Data will be extracted from hospital electronic medical record systems and discharge summaries. Demographic and baseline clinical characteristics of enrolled neonates from both the pre-intervention (historical control) period and the intervention period will be collected, including sex, gestational age, birth weight, admission diagnoses, and comorbidities. In addition to patient-level data, center-level baseline characteristics will be collected for each participating NICU. These characteristics will include local microbiological resistance patterns (antibiograms) and baseline rates of hospital-acquired infections. These data will be used to describe inter-center differences that may influence empirical antibiotic prescribing practices.
2. Process data: collected to calculate process measures, primarily using a standardized Antibiotic Use Case Registry Form. Key variables include a unique study identifier, date and indication for antibiotic initiation, completion of initiation assessment, documentation of the 48–72-h reassessment, and dates and results of blood cultures and other microbiological tests.
3. Outcome data: collected to derive primary and balancing outcomes and extracted from electronic medical records and discharge summaries. These include daily antibiotic administration records (used to calculate antibiotic days), total length of NICU stay, dates and diagnostic basis of LOS, dates and causes of death, and records of antibiotic re-initiation following discontinuation.

#### 2.8.2 Data collection and reporting workflow

A “center-based collection with monthly centralized reporting” model is employed:

1. Local collection: A designated data manager at each hospital extracts and enters data into a uniform Excel template provided by the steering committee.
2. Monthly reporting and audit: Within the first three working days of each month, data managers complete data entry for all eligible cases from the preceding month and submit encrypted Excel files to the regional steering committee. The committee's data management team conducts completeness and logical consistency checks within 1 week (e.g., alignment between antibiotic days and length of stay, internal consistency of calculated indicators). Identified discrepancies are resolved through direct communication with the respective center. After verification, data are aggregated and locked for monthly analysis.

#### 2.8.3 Data quality assurance and security

To ensure data quality, the following measures are implemented:

1. Standardized templates and training: prior to study initiation, all data managers receive standardized training on data definitions, entry procedures, and quality requirements.
2. Source verification and periodic audits: in addition to electronic audits, clinical experts appointed by the regional steering committee conduct quarterly on-site or remote audits at participating centers. Random samples of original medical records are reviewed to verify consistency between registry data and source documentation, and audit findings are incorporated into quality feedback.
3. Data security and confidentiality: all data files are encrypted during transmission and storage. Neonatal information is de-identified using unique study identifiers. The aggregated analytical database is securely maintained by the regional steering committee. Upon study completion, all data will be archived in accordance with applicable regulations.

### 2.9 Statistical analysis

To rigorously evaluate whether the QI intervention achieves its intended goals, complementary analytical approaches will be used to establish a coherent chain of evidence. Statistical process control charts (*P*-charts) will first be used to monitor temporal variation in antibiotic use and to distinguish common-cause variation from special-cause variation potentially associated with the intervention. Special-cause variation observed after implementation will be interpreted as process-level evidence supporting intervention-associated change, particularly when accompanied by improved protocol adherence. Segmented regression ITS analysis will then be used to provide formal statistical inference by estimating the pre-intervention baseline trend, the immediate post-intervention level change, and the change in monthly trend during the intervention period. The overall effectiveness of the QI project will be assessed by integrating primary outcomes, process measures, and balancing measures. The intervention will be considered to have achieved its intended goals if reductions in the antibiotic use rate and/or AUR are observed, accompanied by improved protocol adherence, without concurrent increases in the incidence of late-onset sepsis, in-hospital mortality, or the antibiotic re-initiation rate.

#### 2.9.1 Descriptive analysis

Demographic characteristics of neonates and patterns of antibiotic use are summarized across study phases using descriptive statistics.

#### 2.9.2 Time series analysis

Monthly aggregated data are used as the analytical unit. Statistical process control P charts are applied to assess temporal changes in antibiotic use rates and to identify special-cause variation based on standard run chart rules.

#### 2.9.3 Interrupted time series analysis

Segmented regression models are used to perform ITS analyses for primary outcome measures, estimating both immediate changes in level following intervention implementation (level change) and changes in trend over time before and after the intervention (slope change). Effect estimates are reported with 95% confidence intervals.

#### 2.9.4 Adjustment for center-level variation

To account for potential center-level heterogeneity, center-specific baseline characteristics, including local antibiograms and baseline hospital-acquired infection rates, will be considered in stratified analyses. Intervention adherence rate will also be summarized at the center level and incorporated into the interpretation of between-center differences in observed intervention effects. Where appropriate, mixed-effects models or sensitivity analyses will be performed to evaluate the robustness of intervention effect estimates after accounting for participating centers and relevant center-level factors.

## 3 Discussion

This protocol explores strategies for optimizing antibiotic prescribing in resource-limited regional medical centers. Although global evidence regarding ASPs in NICUs is expanding, existing success stories are predominantly concentrated in elite teaching hospitals in high-income countries, which often rely on sophisticated rapid pathogen diagnostics and dedicated multidisciplinary teams (25, 26). In contrast, within the extensive network of municipal-level hospitals in China, antibiotic utilization rates have remained persistently high due to relatively constrained diagnostic resources and the prevalence of defensive medical practices in clinical settings (14, 27). This study uses a multicenter ITS design to evaluate the potential of a structured ASP intervention to reduce NICU antibiotic use while maintaining safety. Validating this localized intervention strategy will not only address the antibiotic burden in the Suqian region but also provide critical empirical evidence for antimicrobial stewardship pathways in resource-limited environments.

The proposed intervention components are designed to systematically optimize stewardship workflows through multi-level synergy. At the structural level, we established a two-tier governance framework—comprising a regional steering committee and hospital-level core implementation teams—to ensure the unified dissemination and localized execution of standardized protocols and decision-support tools. This structure, supported by monthly collaborative meetings, facilitates continuous data feedback and peer review, thereby ensuring core implementation fidelity across multiple centers. At the procedural level, the intervention centers on the standardization of clinical decision-making. The EOS Risk Assessment and Antibiotic Management Clinical Pathway, developed based on national evidence-based consensus (23), utilizes risk stratification to clarify indications for antibiotic initiation, reducing overuse driven by ambiguous clinical interpretations. Furthermore, the mandatory 48–72-h reassessment mechanism establishes a critical “decision checkpoint,” requiring clinicians to actively re-evaluate treatment plans based on emerging evidence, providing institutional support for minimizing unnecessary treatment prolongation.

Regarding the decision-making environment, previous studies have highlighted the high prevalence of defensive medical behaviors in Chinese clinical practice, often linked to strained patient-physician relationships and concerns regarding medical litigation (27–29). In resource-limited contexts lacking rapid, high-precision diagnostics, clinicians may prefer empirical antibiotic therapy as a risk-aversion strategy rather than relying solely on evidence-based indications. By providing standardized clinical pathways and structured decision support, this study offers clinicians clear, procedural frameworks for judgment, which may help mitigate the tendency toward excessive empirical prescribing driven by diagnostic uncertainty or legal concerns at the institutional level.

Globally, the vast majority of neonates receive care in regional centers similar to those in Suqian rather than in top-tier academic institutions. In such settings, where laboratory diagnostic capacity is relatively limited, the assessment of infection is often accompanied by higher uncertainty, and clinical practice is more prone to rely on empirical antibiotics for risk control (30). Consequently, the simple introduction of complex diagnostic technologies or educational guidelines is often insufficient to achieve sustained impact in daily practice. In contrast, a QI strategy that is clearly structured, embeddable into existing workflows, and does not significantly increase workload may better meet the practical needs of such environments. By simultaneously implementing and evaluating this intervention across multiple regional NICUs, this study provides a practical framework for other regions with similar resource profiles to explore stewardship improvement pathways.

Methodologically, the multicenter ITS design evaluates trend changes while controlling for time-related confounding (31). Moreover, the concurrent focus on primary, balancing, and process measures ensures a comprehensive assessment of the intervention's potential effectiveness and safety. However, this study has limitations. As a real-world QI initiative, the influence of unmeasured confounders cannot be entirely excluded. Additionally, the study's impact depends partly on the consistency of protocol execution and data quality across centers. Although the 12-month intervention period is sufficient for evaluating early level and trend changes within an interrupted time series framework, it may still be insufficient to fully assess the long-term sustainability of the intervention effect or to determine whether observed improvements can be sustained over time under routine practice conditions. Longer follow-up will therefore be important in future work. In addition, although AUR and DOT are widely used and highly comparable outcome measures in neonatal antimicrobial stewardship research, they do not fully capture differences in antibiotic spectrum intensity. Spectrum-based metrics such as antibiotic spectrum index or days of antibiotic spectrum coverage were not included in the present study because currently available scoring systems have not been adequately adapted or validated for neonatal populations, particularly with respect to common neonatal pathogens such as group B streptococci and coagulase- negative staphylococci (32, 33). Finally, this protocol does not include a formal cost-effectiveness analysis, which may be an important consideration for future large-scale implementation.

## 4 Conclusion

This study proposes and systematically evaluates a multicenter antimicrobial stewardship QI program tailored for regional NICUs, addressing the persistent challenge of high antibiotic utilization in relatively resource-limited settings. By establishing a multi-tier governance structure, standardizing key clinical decision-making processes, and implementing continuous monitoring and feedback mechanisms, this study provides a pragmatic and reproducible pathway for optimizing neonatal antibiotic use in real-world clinical environments. Our approach highlights that through workflow restructuring and organizational synergy, antimicrobial stewardship can be effectively standardized without the necessity of highly complex technologies or substantial additional resource investment. The implementation experience gained from this study will serve as a reference for NICU stewardship interventions in similar resource contexts and lay the foundation for future evaluations of long-term impact and generalizability.
