# Supplementary material for: Antimicrobial stewardship in neonatal intensive care units in resource-limited regions of China: a protocol for a multicenter, before-and-after, interrupted time series quality improvement study
Source: Front Public Health. 2026 Jun 2;14:1837412. doi: 10.3389/fpubh.2026.1837412 (PMC13268985; doi:10.3389/fpubh.2026.1837412)
Supplement: Supplementary file 1 [file Data_Sheet_1.docx]

Supplementary Material

**Table S1**. Criteria for abnormal nonspecific laboratory findings

| Test | Criteria for abnormality |
| --- | --- |
| WBC (white blood cell count) | Presence of any of the following:  (1) WBC ≥ 25 × 10⁹/L at 6 hours to 3 days after birth;  (2) WBC ≥ 20 × 10⁹/L after 3 days of life;  (3) WBC < 5 × 10⁹/L at any postnatal age;  (4) Absolute neutrophil count < 1 × 10⁹/L at any postnatal age. |
| I/T ratio (immature-to-total neutrophil ratio) | Presence of any of the following:  (1) I/T ratio ≥ 0.16 from birth to 72 hours of life;  (2) I/T ratio ≥ 0.12 after 72 hours of life. |
| PLT (platelet count) | PLT ≤ 100 × 10⁹/L. |
| CRP (C-reactive protein) | Presence of any of the following:  (1) CRP > 3 mg/L within 6 hours after birth;  (2) CRP > 5 mg/L at 6–24 hours after birth;  (3) CRP ≥ 10 mg/L after 24 hours of life. |
| PCT (procalcitonin) | PCT may show physiological elevation within the first 72 hours of life. A PCT value > 0.5 ng/mL after 72 hours of life is considered abnormal. |

**Note:** These laboratory parameters are nonspecific markers of infection. Procalcitonin levels may increase physiologically during the first 72 hours after birth and should be interpreted with caution during this period. All laboratory findings should be evaluated in conjunction with clinical presentation and perinatal risk factors.

**Table S2**. Diagnostic criteria for neonatal sepsis

| Category | Diagnostic criteria |
| --- | --- |
| Suspected sepsis | **EOS:** ≤72 hours of life, diagnosis can be made if **any one** of the following is present:  (1) Clinical signs suggestive of infection;  (2) Presence of major risk factors for EOS.  **LOS:** >72 hours of life, diagnosis can be made when clinical signs of infection are present. |
| Clinical sepsis | Presence of clinical signs of infection and at least one of the following:  (1) ≥2 positive nonspecific laboratory tests;  (2) Cerebrospinal fluid findings consistent with purulent meningitis;  (3) Detection of pathogen DNA in blood or cerebrospinal fluid. |
| Confirmed sepsis | Presence of clinical signs of infection and a positive culture from blood, cerebrospinal fluid, or another normally sterile body site. |

**Note:** EOS, early-onset sepsis; LOS, late-onset sepsis.
